# Role of IL-32 in RA pathology and potential as a drug target

**DOI:** 10.3389/fimmu.2026.1696081

**Published:** 2026-01-28

**Authors:** Hongliang Zhang, Qingyuan Chen, Hui Yu, Mingzi Zhu, Xiaoqing Zhang, Xin Fu, Songquan Wu, Guangli Wang

**Affiliations:** 1 Center of Disease Immunity and Intervention, College of Medicine, Lishui University, Lishui, China; 2 Zhejiang Xinchang Agricultural Development Co., Ltd., Lishui, Zhejiang, China; 3 Department of Pharmacy, Lishui hospital of traditional Chinese medicine, Lishui, Zhejiang, China

**Keywords:** cytokines, fibroblast-like synoviocytes, IL-32, macrophages, rheumatoid arthritis

## Abstract

Interleukin-32 (IL-32) is a cytokine involved in a broad repertoire of immunopathological events across both physiological and disease contexts, encompassing immune modulation, inflammatory amplification, and tumor initiation and progression. IL-32 propagates systemic inflammatory cascades by vigorously inducing pivotal mediators such as TNF-α and IL-17. Consequently, its dysregulated expression has been implicated in diverse disorders, and it has emerged as a tractable therapeutic target. Rheumatoid arthritis (RA) is an autoimmune disease characterized by persistent inflammatory synovitis that inexorably erodes articular cartilage and subchondral bone, resulting in debilitating pain, swelling, joint stiffness, and irreversible functional decline. IL-32 is markedly upregulated in the RA synovium, synovial fluid, and peripheral blood, and its abundance is positively correlated with clinical indices of disease activity. Mechanistically, IL-32 induces several cytokines in RA especially TNF-α and IL-17—two master cytokines of RA pathogenesis—thereby amplifying synovial inflammation, osteoclastogenesis, and subsequent joint destruction. Preclinical studies have demonstrated that genetic or pharmacologic inhibition of IL-32 attenuates experimental arthritis severity, underscoring its therapeutic potential. Herein, we provide a comprehensive, up-to-date review of the current understanding of IL-32 biology in RA and its translational implications.

## 1 Introduction

Interleukin-32 (IL-32) was first found in activated natural killer (NK) cells and termed as NK4 in 1992 (1). Its proinflammatory properties were then established in 2005, when it was shown to induce several key cytokines: human tumor necrosis factor-alpha (TNF-α) and IL-8 in monocytes, and murine TNF-α and macrophage inflammatory protein-2 (MIP-2) in macrophages (2). On the basis of this function, it was reclassified as interleukin-32 (IL-32). IL-32 transcripts are widely expressed throughout human tissues and organs, with notably higher levels in immune cells than in non-immune tissues (3, 4). IL-32 functions primarily intracellularly; however, in some contexts, it can also be secreted via exosomes, extracellular vesicles, or even from apoptotic cells (5–7). In addition to inducing inflammation, IL-32 also exhibits potent antiviral activity (8). Recently, IL-32 has emerged as a biomarker in multiple pathologies including inflammatory diseases and cancer (9–13).

Rheumatoid arthritis (RA) is a chronic autoimmune disease characterized by persistent inflammatory synovitis, which leads to the classic symptoms of joint pain, swelling, morning stiffness, and progressive loss of function (14, 15). The disease can develop at any age, peaking between 35 and 60 years, with women being affected 2 to 3 times more often than men are affected (16). The global prevalence of RA is approximately 0.5%. Although the precise etiology of RA remains elusive, multiple interconnected risk determinants have been identified. Genetic susceptibility is strongly conferred in RA by alleles such as HLA-DR4 and HLA-DR1 (17). A range of infections, including *Epstein–Barr* virus, *Mycoplasma*, and *Streptococcus*, may precipitate autoimmunity in RA (18, 19). In addition, hormonal influences, particularly estrogen fluctuations, underpin the marked female predominance (20, 21). Finally, environmental and lifestyle exposures—cigarette smoking, obesity, silica dust, periodontal disease, and gut dysbiosis—collectively modulate RA risk (22). Currently, the diagnosis of RA hinges on six weeks or more of symmetric swelling and dawn stiffness in the small joints, corroborated by rheumatoid factor (RF), anti- cyclic citrunilated peptides (CCP) antibodies, acute-phase reactants and ultrasound or magnetic resonance imaging (MRI) (23, 24). In most cases, RA therapy begins with methotrexate and traditional disease-modifying antirheumatic drugs (DMARDs), but when inflammation is reversed, TNF-α blockers, IL-6 antagonists, Janus kinase (JAK) inhibitors, and, ultimately, prosthetic joints standing ready are used. Nonsteroidal anti-inflammatory drugs (NSAIDs), brief corticosteroid bridges, disciplined exercise, weight control, and a final, firm farewell to cigarettes complete the armamentarium (25).

The expression level of IL-32 is markedly increased in RA serum, peripheral blood cells, synovium, and synovial fluid (SF), and its abundance is positively correlates with clinical indices of disease activity. Various factors induce IL-32 expression in RA, including TNF-α, IL-17, and IFN-γ. These findings suggest that IL-32 plays a key role in RA. Injection of IL-32 into mouse joints resulted in inflammatory cell infiltration and bone damage. Mechanistically, IL-32 robustly upregulated inflammatory cytokines such as TNF-α, IL-17, IFN-γ, IL-4, IL-12, and IL-6 to exacerbate inflammation in RA. IL-32 also exacerbates bone destruction by triggering osteoclastogenesis and subsequent joint destruction. Most importantly, preclinical studies have demonstrated that genetic or pharmacologic inhibition of IL-32 attenuates experimental arthritis severity, underscoring its therapeutic potential. Collectively, these findings underscore IL-32 as a pivotal cytokine implicated in the pathogenesis of RA. Herein, we provide a comprehensive, up-to-date review of the current understanding of IL-32 biology in RA. Delineating future research trajectories and their translational ramifications for clinical application.

## 2 IL-32: structure and signaling

**Figure 1:**
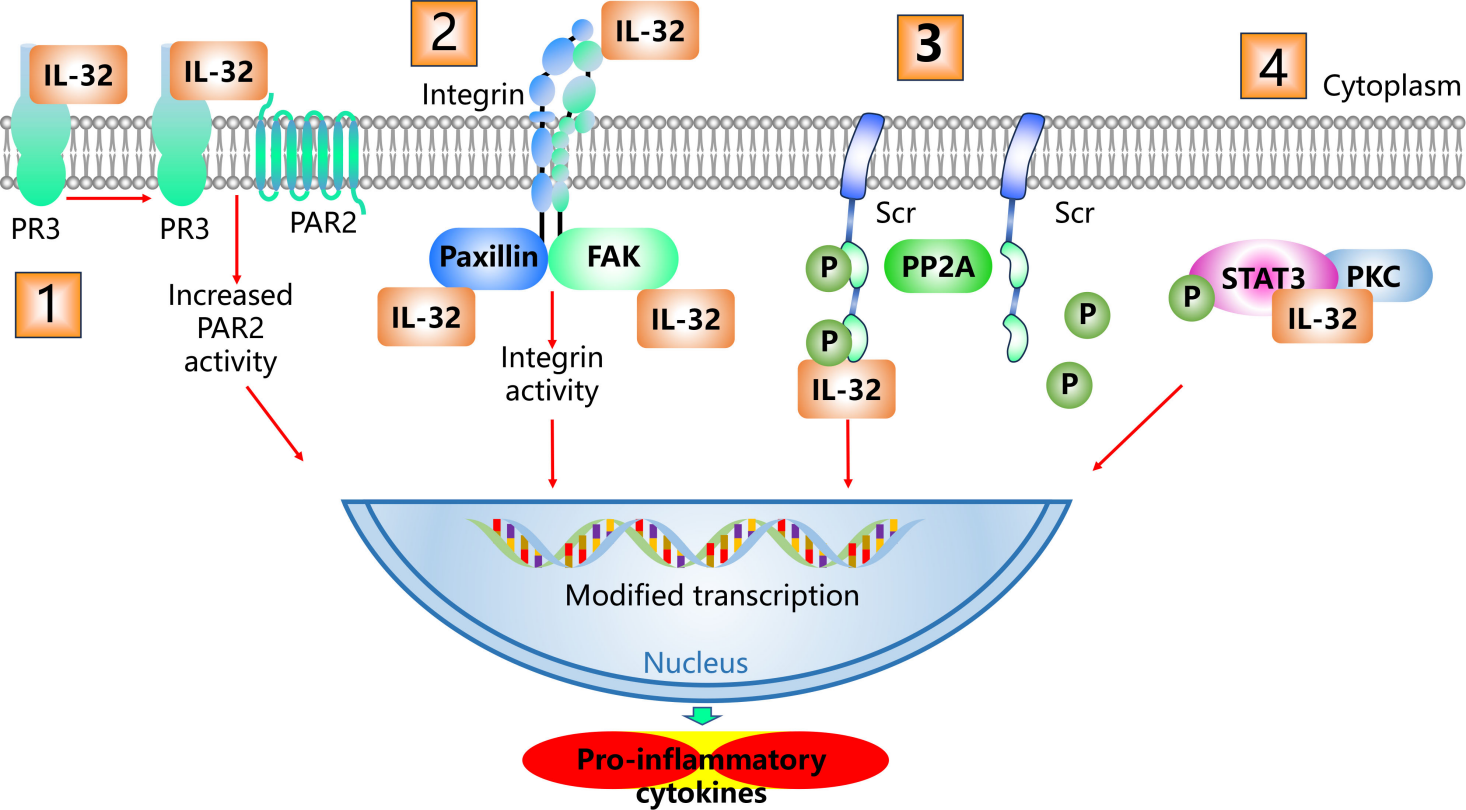
IL-32 signaling pathway. ① Extracellular IL-32 interacts with PR3 and activates the PAR2 Signaling axis. ② Extracellular IL-32 interacts with integrins and activates the FAK/paxillin complex. ③ Intracellular IL-32 interacts with Src. ④ Intracellular IL-32 interacts with PKC and STAT3. IL-32 acts on these pathways to drive the transcription and expression of various proinflammatory cytokines.

The IL-32 gene, comprising at least eight exons, is located on human chromosome 16p13.3 and is evolutionarily conserved in higher mammals (e.g., equine, porcine, and ovine species) (2, 26). Alternative splicing of the IL-32 gene yields nine confirmed IL-32 variants—α, β, γ, δ, ϵ, ζ, η, θ, and sm (2, 26–28). Genetically cloned and purified recombinant IL-32 isoforms all possess biological activities that induce various proinflammatory cytokines and chemokines. Among these isoforms, both IL-32β and IL-32γ exhibit more potent activity than do IL-32α and IL-32δ (29). IL-32γ—the longest isoform corresponding to the original NK4 transcript—demonstrates superior bioactivity, inducing significantly greater TNF-α production than other variants do (29). IL-32γ possesses 234 amino acid residues and all 11 protein domains. These distinct domain architectures enable different IL-32 isoforms to interact with specific intracellular and extracellular molecules, thereby defining their unique functions (30). Notably, while rodents (e.g., mice) lack an orthologous IL-32 gene (26), their cells remain responsive to IL-32 stimulation, eliciting proinflammatory cytokine production (2, 31). Although the X-ray/NMR structure of IL-32 remains unsolved, software prediction revealed secondary and tertiary structures with coils and α-helixes but no β sheets (32). Proteinase 3 (PR3) may function as a receptor for IL-32. Both IL-32α and IL-32γ can bind to PR3 and be internalized to initiate intracellular signaling (33, 34). IL-32α can bind to both urinary PR3 (K_d_ = 2.65 × 10^−8^ M) and neutrophil PR3 (K_d_ = 1.2 × 10^−8^ M) (33). IL-32γ binding enhances the serine protease activity of PR3, triggering subsequent PAR2 activation. This IL-32γ–PAR2 signaling axis drives the expression of TNF-α and IFN-β (35). However, this axis is unlikely to be the primary signaling pathway for IL-32, as cell types lacking PR3 or PAR2 expression remain responsive to IL-32. Integrin represents another potential receptor for IL-32. IL-32 contains Arg-Gly-Asp (RGD) motifs, which facilitate its extracellular binding to various integrins. This binding activates intracellular signaling through the focal adhesion kinase (FAK)/paxillin complex and its downstream pathways. Intriguingly, IL-32 has been shown to physically interact with both paxillin and FAK intracellularly (32). These findings suggest that IL-32 acts on integrins to modulate the adhesion and migration of target cells. Furthermore, hypoxia-induced IL-32β was found to stimulate Src activation by inhibiting its dephosphorylation (7). IL-32𝛼 triggers signal transducer and activator of transcription (STAT) 3 Ser727 phosphorylation by creating a trimeric complex with protein kinase C (PKC) 𝜖 and STAT3, which promotes IL-6 production (36) (**Figure 1**). IL-32 also activates canonical cytokine signaling pathways, such as nuclear factor-kappa B (NF-κB) and p38 mitogen-activated protein kinase (p38MAPK), to produce TNF-α and other chemokines (2, 37).

## 3 Induction of IL-32 in RA

While constitutive IL-32 expression does occur, it is more commonly upregulated in response to proinflammatory stimuli, pathogens, and cellular stressors such as hypoxia (38–40). Ligands for TLR2, TLR3, and TLR4, as well as the cytokines IFN-γ and TNF-α, induce the expression of IL-32 in RA fibroblast-like synoviocytes (FLSs) (41). When RA FLSs were stimulated with IFN-γ, IL-32β, γ, and δ transcripts were upregulated in a dose-dependent manner, a response that significantly exceeded that of normal or OA FLSs. In contrast, TNF-α stimulation induces the expression of four isoforms (IL-32α, β, γ, and δ) in RA FLSs (42). Interestingly, unlike the secreted IL-32β, γ, and δ isoforms, TNF-α-induced IL-32α was detected intracellularly but was not released. Furthermore, the mechanisms of TNF-α-induced IL-32 expression are time dependent. Short-term treatment (hours) activates IL-32 independently of DNA demethylation, whereas prolonged treatment (days) induces DNA demethylation at the IL-32 promoter and a CpG island. This long-term effect is mediated through a mechanism dependent on both ten-eleven translocation (TET) family enzymes and NF-κB (43). The hypomethylated state of IL-32 regulatory regions was maintained for several weeks, resulting in persistently elevated IL-32 expression independent of continuous TNF-α stimulation (43). In RA FLSs, specific pathogen-associated molecular patterns (PAMPs) induce distinct IL-32 isoforms: BLP (Bacterial Lipoprotein) and poly (I:C) triggered the expression of IL-32β, γ, and δ transcripts, whereas LPS induced only the β and δ isoforms. Furthermore, synergistic stimulation with IFN-γ and TNF-α strongly induced IL-32 mRNA expression in an interferon regulatory factor-1 (IRF-1) -dependent manner (42). IL-17 markedly upregulated IL-32 in RA FLSs via NF-κB and phosphatidylinositol-3-kinase (PI3-K) in the RA synovium (44). In addition, enolase-1 (ENO1), a glycolytic enzyme, induces IL-32 expression in RA peripheral blood mononuclear cells (PBMCs) in an NF-κB- and p38 MAPK pathway -dependent manner (45). For secretion, IL-32γ is predicted to harbor a specific transmembrane domain that anchors it in the membrane and a PR3 (membrane expression elevated in RA) cleavage site that enables its proteolytic release into the extracellular milieu (32, 46). Taken together, PAMPs (BLP, poly(I:C), and LPS) and various cytokines (IFN-γ, TNF-α, and IL-17) induce IL-32 expression in RA, and this upregulation of IL-32 may contribute to RA pathogenesis.

## 4 The role of IL-32 in RA pathology

### 4.1 IL-32 triggers inflammatory cytokine release to propagate synovitis

IL-32 is a well-established proinflammatory cytokine that drives robust induction of proinflammatory cytokines such as IL-1α, IL-1β, IL-6, TNF-α, IL-8, and multiple chemokines through coordinated activation of the NF-κB, p38 MAPK, and activating protein-1 (AP-1) signaling pathways (47–49). Therefore, IL-32 may activate inflammatory cytokines to propagate synovitis in RA.

Compared with phosphate-buffered saline (PBS) injection, the injection of IL-32 into both knee joints of a collagen-induced arthritis (CIA) model resulted in significantly greater inflammation (50). These findings suggest that IL-32 contributes to the development of inflammatory synovitis in RA. In fact, this IL-32 treatment significantly induced the production of proinflammatory cytokines such as IL-1β, TNF-α, IL-18, and IFN-γ (50). Moreover, the expression of the chemokine receptors CCL2 and CXCL9 and the chemokines CCR2 and CCR5 is also significantly induced by IL-32 in the joints (50). These findings suggest that IL-32 induces the expression of proinflammatory cytokines and chemokines to promote joint inflammation in RA. Moreover, IL-32γ robustly upregulated IL-6 and IL-8 expression in FLSs isolated from RA patients, accompanied by pronounced phosphorylation of Erk1/2 and activation of AP-1 (51). Pharmacologic inhibition revealed that blockade of extracellular signal- regulated kinase (ERK) 1/2—but not AP-1—abolished IL-6 and IL-8 transcription, establishing that IL-32γ drives the expression of these cytokines in an Erk1/2-dependent manner (51). In addition, IL-32γ synergizes with ligands of TLR-2 and NOD2 to amplify the production of proinflammatory cytokines and chemokines, thereby exacerbating joint inflammation and cartilage destruction during destructive arthritis. This synergy is potentiated by the ability of IL-32γ to upregulate TLR-2 and NOD2 expression in FLSs (52). IL-32 also markedly upregulated the production of thymic stromal lymphopoietin (TSLP), a proinflammatory cytokine with multifaceted roles in RA pathogenesis, in both THP-1 cells and primary human blood monocytes. This induction proceeded through simultaneous activation of caspase-1 and the NF-κB signaling pathway (53).

IL-32 and TNF-α mutually interact in RA. Intra-articular injection of human IL-32γ into mice resulted joint swelling, inflammatory cell infiltration, and cartilage damage. However, genetic knockout of TNF-α significantly reduces inflammatory cell influx and joint swelling in this IL-32γ -injected RA model (54). These findings suggest that TNF-α is indispensable for IL-32 -induced arthritis. In addition, the transfer of IL-32β-secreting CD4+ T cells into a CIA model resulted in exacerbated arthritis, whereas the antibody-mediated blockade of TNF-α significantly ameliorated the severity of this IL-32β-induced disease (31). These findings establish that the pathogenicity of IL-32 in RA is, at least in part, mediated by TNF-α. Moreover, the protein levels of IL-32 in RA joints strongly correlate with those of TNF-α (54). These findings suggest that there is a mutual interaction between IL-32 and TNF-α. In fact, recombinant IL-32γ induces TNF-α secretion from human PBMCs and mouse macrophages in a dose-dependent manner (55). Administration of anti-TNF-α antibodies to RA patients led to a significant decrease in synovial IL-32 protein expression (56). Concurrently, TNF-α stimulation robustly upregulated IL-32γ expression in cultured FLSs (56, 57), monocyte-derived dendritic cells, and T cells (31). TNF-α induces IL-32 expression via the Syk/PKCδ/JNK signaling pathway, as demonstrated by the significant inhibition observed when both specific inhibitors and siRNAs targeting these components were used (57, 58). Taken together, these findings suggest that a key autoamplification loop in inflammation is driven by IL-32, which potently stimulates TNF-α production. TNF-α then acts to perpetuate IL-32 expression, leading to a sustained inflammatory response (56). The self-sustaining feedback circuit between IL-32 and TNF-α has the potential to amplify pathological inflammation, thereby promoting the establishment of chronic inflammatory disease (59). Overall, IL-32 contributes to the development of inflammatory synovitis in RA by activating proinflammatory cytokines of the innate immune system.

### 4.2 IL-32 triggers cellular responses to propagate synovitis

In addition to inducing cytokines and chemokines, IL-32 also induces inflammatory cells in RA. IL-32γ treatment of immature dendritic cells (DCs) significantly induced the expression of maturation and activation associated molecules (MHC class II, CD86, and CD40) and proinflammatory cytokines (IL-6, IL-1β, TNF-α, IL-12, IL-23, and IL-27) on DCs in a dose-dependent manner (37). These findings suggest that IL-32 promotes DC maturation. Moreover, the increased IL-12 and IL-6 levels in IL-32 γ-treated DCs are known as Th1- and Th17-polarizing cytokines. Further studies revealed that coculture of IL-32 γ-treated DCs with CD4 ^+^ T cells led to increased Th1 and Th17 responses, as increased IFN-γ- and IL- 17-secreting T-cell populations were identified, respectively. The adoptive transfer of IL-32 γ-stimulated DCs into ovalbumin-sensitized mice also confirmed this (37). These findings suggest that IL-32 promotes DC maturation to induce Th1 and Th17 cells to exacerbate inflammation.

Macrophages also play a critical role in the pathogenesis of RA. Macrophages possess functional plasticity, which allows them to differentiate into various phenotypes and act as central effector cells in RA (60). IL-32 drives monocyte-to-macrophage differentiation through a nonapoptotic process that is dependent on caspase-3, thereby amplifying the inflammatory response (48). Moreover, TSLP is required for the IL-32-induced differentiation of monocytes into macrophage-like cells (53). These findings suggest that IL-32 induces macrophage to promote inflammation in RA. In addition, IL-32 also drives NK cell activation in RA. The expression of NKp46, a marker of NK cell activation, is significantly increased in IL- 32-treated CIA synoviocytes (50). These findings suggest that IL-32 also increases the number of NK cells in RA to amplify inflammation. Taken together, these findings indicate that IL-32 promotes inflammatory cell maturation and activation to trigger inflammation in RA.

### 4.3 IL-32 induces bone and cartilage destruction

**Figure 2:**
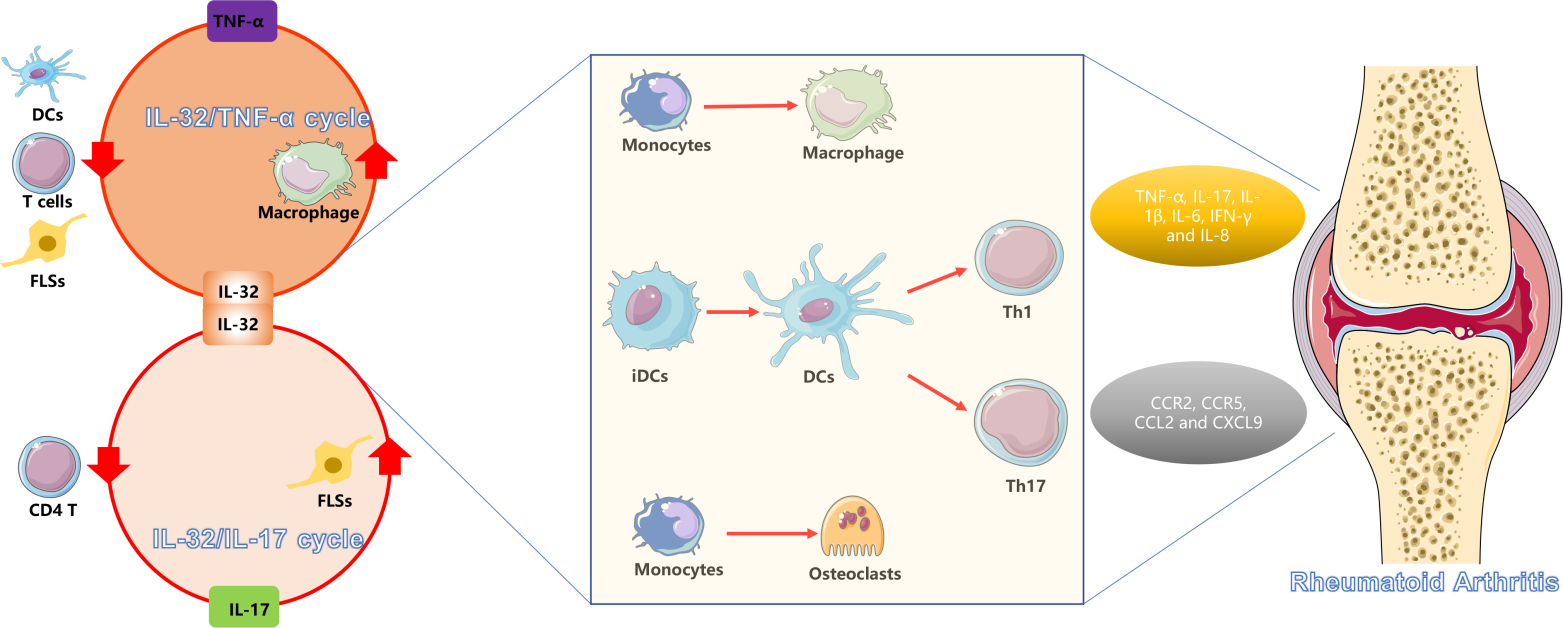
Schematic representation of the pathogenic role of IL-32 in RA. The left panel are two primary positive feedback loops of IL-32 in RA: the IL-32/TNF-α cycle and the IL-32/IL-17 cycle. These cycles facilitate interactions among DCs, T cells, macrophages, and FLSs. As shown in the center panel, IL-32 promotes the differentiation of monocytes into macrophages and osteoclasts, as well as the maturation of iDCs into mature DCs, which subsequently drive Th1 and Th17 cell differentiation. This cascade results in the excessive production of pro-inflammatory cytokines and chemokines, ultimately leading to synovial inflammation and bone destruction in RA.

In addition to driving synovial inflammation, IL-32 also directly contributes to bone erosion and cartilage destruction in RA. RA patients who have bone destruction have higher serum levels of IL-32 than those with no or mild bone destruction (61), suggesting that IL-32 may contribute to bone destruction in RA patients. In fact, injection of IL-32 in a CIA model significantly increased bone destruction compared with that in the PBS group (50). Specifically, IL-32 potently converts adherent PBMCs into multinucleated TRAcP^+^VNR^+^ cells that display canonical osteoclast markers and likewise directs purified CD14 + monocytes along the osteoclastic lineage (62, 63). However, IL-32 is insufficient on its own to advance these nascent osteoclasts to the functionally mature, bone-resorbing stage (62, 63). In the presence of soluble receptor activator of nuclear factor-κB ligand (RANKL), IL-32γ drives both osteoclast formation and bone resorption to a significantly greater extent than does IL-17—a cytokine long recognized for its ability to trigger osteoclastogenesis from monocytes and to amplify RANKL production by RA FLSs (63). These findings establish that IL-32γ is a potent, RANKL-dependent amplifier of osteoclastogenesis (64). Specifically, IL-32γ inhibits osteoprotegerin (OPG) expression, leading to an increased RANKL: OPG ratio in FLSs, which promotes osteoclastogenesis in the RA joint (64). There is a mutual interaction between IL-32 and IL-17 in RA. IL-32 elicits IL-17 production by CD4 + T cells, while IL-17 reciprocally upregulates IL-32 expression in RA FLSs. This reciprocal crosstalk amplifies RANKL-dependent osteoclast differentiation and bone resorption, establishing a synergistic IL-32/IL-17 circuit that potently drives pathological bone loss (44) (**Figure 2**).

### 4.4 IL-32 induce anti-inflammatory effects in RA

Like most other factors, IL-32 also has another effects on RA. IL-32 triggers the release of IL-4 and IFN-γ — well-characterized suppressors of osteoclast formation and activation — underscoring its nuanced, dual-edged role in the osteolytic milieu of RA (62). IL-32γ induces IL-10 production by DCs (37). IL-10 is an anti-inflammatory cytokine that is elevated in both the serum and the synovial fluid of individuals with RA (65). Neutralization of IL-10 leads to elevated expression of TNF-α and IL-1β in RA (66), and the addition of exogenous IL-10 tends to be effective in treating RA (67). These findings suggest that IL-32 γ-induced IL-10 may alleviate RA progression. Interestingly, IL-32γ can be spliced into IL-32β, which also induces IL-10 production (68). IL-32 β-overexpressing mice were generated genetically, followed by stimulation with a collagen antibody and lipopolysaccharide, and the results revealed that IL-32β significantly reduced the production of proinflammatory cytokines, alleviating inflammatory arthritis (69). Transforming IL-32γ to IL-32β alleviated IL-32 γ-induced inflammation and acted as a safe control for chronic inflammation (70). In addition, IL-32 redirected DC differentiation towards macrophages induced by GM-CSF/IL-4 (48). In summary, IL-32 can induce anti-inflammatory effects in RA.

## 5 Clinical relevance of IL-32 in RA

IL-32 mRNA was significantly upregulated in the PBMCs of patients with active RA, compared with both healthy controls and those with stable disease (71). Furthermore, a significant positive correlation was observed between IL-32 mRNA expression levels and key inflammatory markers such as TNF-α, the erythrocyte sedimentation rate (ESR), C-reactive protein (CRP), RF, and the Disease Activity Score in 28 joints (DAS28) in RA patients (71). Serum IL-32 levels are also significantly elevated in patients with RA compared with healthy controls (61, 72), especially in patients with active RA (71). Serum IL-32 levels are positively correlated across a spectrum of RA features, from specific immune mediators (chemokine C-X-C motif chemokine 13 (CXCL13), TNF-α, and IL-6) and autoantibodies (anti-CCP) to systemic inflammation (ESR and CRP), clinical disease activity (DAS28), and structural damage (X-ray stage and bone destruction) (61). Furthermore, elevated IL-32 expression is a prominent feature of the RA joint, with high levels observed in both synovial tissue and synovial fluid (54, 57). The intensity of IL-32 immunostaining in the RA synovium significantly positively correlated with clinical measures of disease (ESR (synovial inflammation index)) and with the local expression of proinflammatory cytokines (TNF-α, IL-1β, and IL-18) (54). Taken together, these results suggest that IL-32 is elevated in RA patients and may contribute to disease progression. Moreover, the levels of IL-32 in synovial tissue and synovial fluid are greater in RA than in OA (64). Comparative transcriptomic profiling of *in vitro* cultured fibroblasts derived from RA and OA patients demonstrated marked differential expression of cytokine/chemokine genes, with IL-32 exhibiting the most pronounced disparity between the two cohorts (73). Taken together, these results suggest that the expression of IL-32 closely related to RA pathogenesis and that IL-32 is a specific cytokine of clinical value for RA treatment.

## 6 Targeting IL-32 for RA treatment

IL-32 is markedly upregulated in RA and potently exacerbates inflammation and bone destruction; paradoxically, it can also trigger anti-inflammatory circuits. This multifaceted role of IL-32 in RA presents both a challenge and an opportunity for developing novel targeted therapies. Given that IL-32 induction in RA is driven by a range of stimuli (e.g., proinflammatory cytokines, infections, and cellular stress), a logical therapeutic approach is to mitigate IL-32-driven pathology by using pharmacological inhibitors that target these induction signals. For example, targeting ENO1 with inhibitors possibly blocks ENO1-induced IL-32 expression in RA. The activity of IL-32 can also be targeted with antibodies and small-molecule inhibitors. For example, bamboo salt significantly attenuated the production of proinflammatory cytokines and the differentiation of macrophages induced by IL-32 (74). Upregulated IL-32 may then interact with receptors such as PR3/PAR2, PKC, Src, and integrin to induce pathology. Therefore, targeting IL-32 downstream molecules and signaling pathways also promotes IL- 32-targeted treatment in RA. For example, various types of viable PR3 inhibitors can also be used to block IL-32 downstream (75–77). Moreover, these was crosstalk between IL-32 and other cytokines such as TNF-α and IL-17. Coadministration of IL-32 inhibitors with TNF- α- and IL- 17-targeted drugs or replacement of these drugs with IL-32 inhibitors possibly holds promise for better treatment and even overcoming biological resistance in some RA patients. The coadministration of IL-32 inhibitors with conventional synthetic DMARDs also represents a promising strategy for RA treatment in the future.

## 7 Conclusion

Over the past two decades, the use of IL-32 has evolved from a “cytokine-in-search-of-a-function” approach to a nonredundant amplifier of synovial inflammation in RA. IL-32 in RA displays a “three-high” signature: high abundance—significantly elevated in PBMCs, serum, synovial tissue, and fluid versus healthy or stable RA; high specificity—synovial levels of RA are higher than those of OA, and the most divergent transcript in RA-FLSs versus OA-FLSs; and high correlation—positively associated with TNF-α, IL-6, CRP, ESR, DAS28, and radiographic bone damage. Overall, IL-32 is an RA-specific cytokine closely linked to disease activity and progression. Mechanistically, IL-32 promotes inflammation by inducing proinflammatory cytokines (IL-1β, TNF-α, IL-18, IL-6, IL-8, and IFN-γ), and inflammatory cells (NK, DCs, macrophages, Th1, and Th17), which interact with TNF-α. IL-32 promotes osteoclastogenesis, leading to bone and cartilage destruction. Moreover, IL-32 also induces anti-inflammatory effects in RA, indicating that IL-32 is a tractable therapeutic node that operates upstream of multiple pathogenic cascades but downstream of shared genetic risk factors.

Targeting IL-32 for the treatment of RA still faces many challenges in clinical translation. IL-32 exists in multiple splicing isoforms (such as IL-32α, β, and γ), and their functions are not entirely identical—some may even have anti-inflammatory effects. If a drug targets only one of them, compensation by other isoforms may lead to suboptimal efficacy; if broad inhibition is applied, it may interfere with their potential physiological functions and increase the risk of side effects. In addition, IL-32 is believed to function through various cell surface proteins, which makes it difficult to design drugs that precisely block “ligand–receptor” interactions, such as monoclonal antibodies. Since IL-32 lacks classical enzymatic activity or a well-defined receptor binding pocket, designing and discovering small-molecule drugs that can efficiently and specifically block its function is also extremely challenging. Most importantly, the IL-32 gene is unique to humans and other primates and is absent in commonly used laboratory animals such as mice and rats, which makes preclinical studies lacking ideal animal models. Since IL-32 also plays a significant role in antiviral and antibacterial immunity, comprehensively inhibiting IL-32 may increase the risk of infections, particularly opportunistic infections.

To date, the protein structure of IL-32 remains unresolved, resulting in a major obstacle to deciphering its molecular mechanisms. Consequently, short-term efforts to determine its structure represent a critical direction for the field. The IL-32 sequence is poorly conserved across species, but organisms lacking an IL-32 ortholog can still respond to IL-32 stimulation, suggesting the existence of an IL-32-like protein or its receptor. Identifying this potential protein also represents a key research direction for IL-32. In addition, while IL-32 induces a substantial number of cellular events, only a few of them have been confirmed in RA, suggesting that extensive investigations into the role of IL-32 in RA are urgently needed. To provide precise IL- 32-targeted therapy for RA, the specific functions of various IL-32 isoforms in RA should be elucidated, and the expression of IL-32 should also be tested before treatment. Exploring combination therapies that target IL-32 and DMARDs is also an important future research direction, particularly for highly refractory RA patients. This approach may yield a synergistic effect, achieving deeper inflammation control. At the same time, it is necessary to identify biomarkers for predicting treatment efficacy to enable precise medication.
